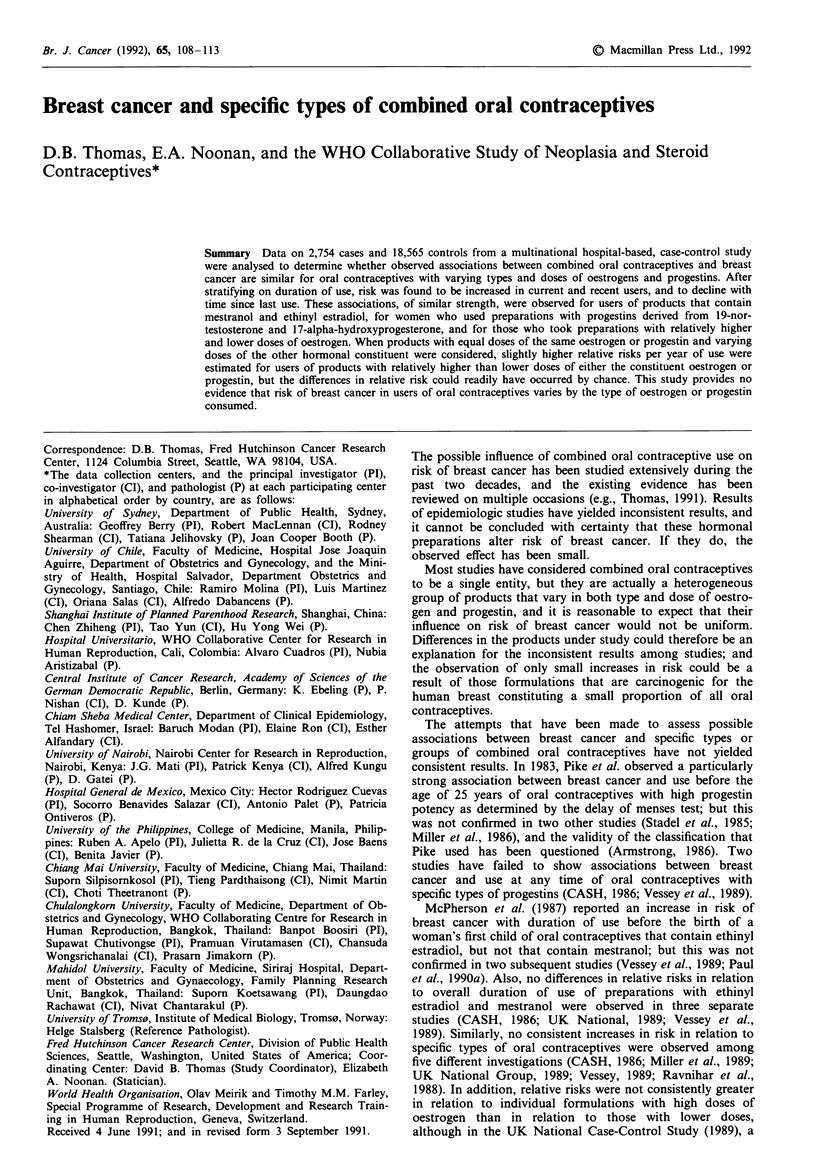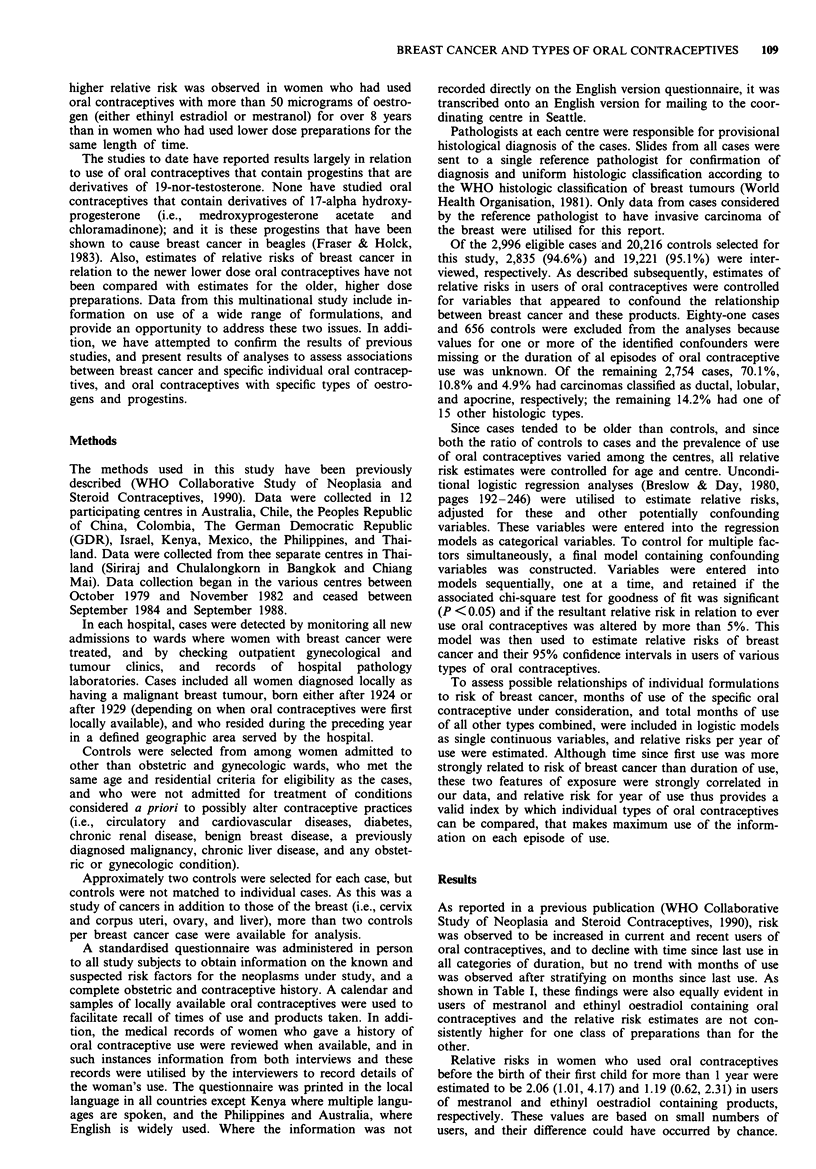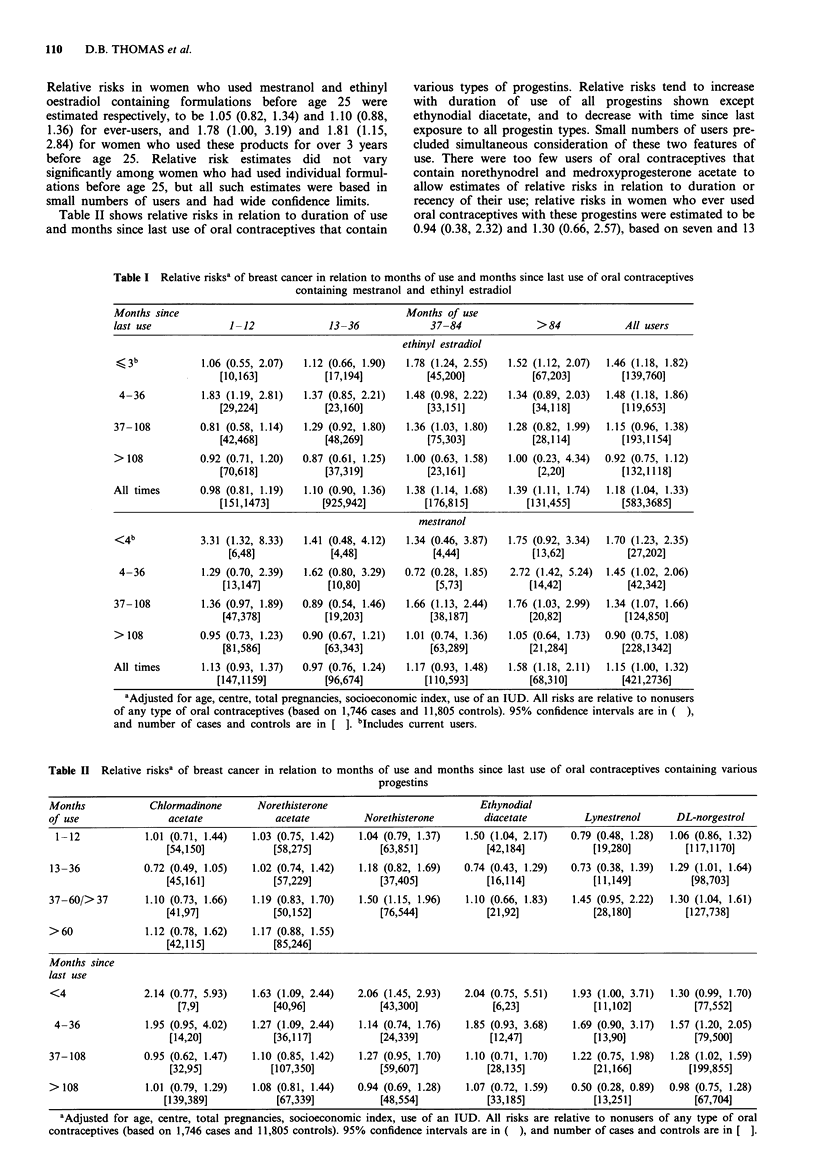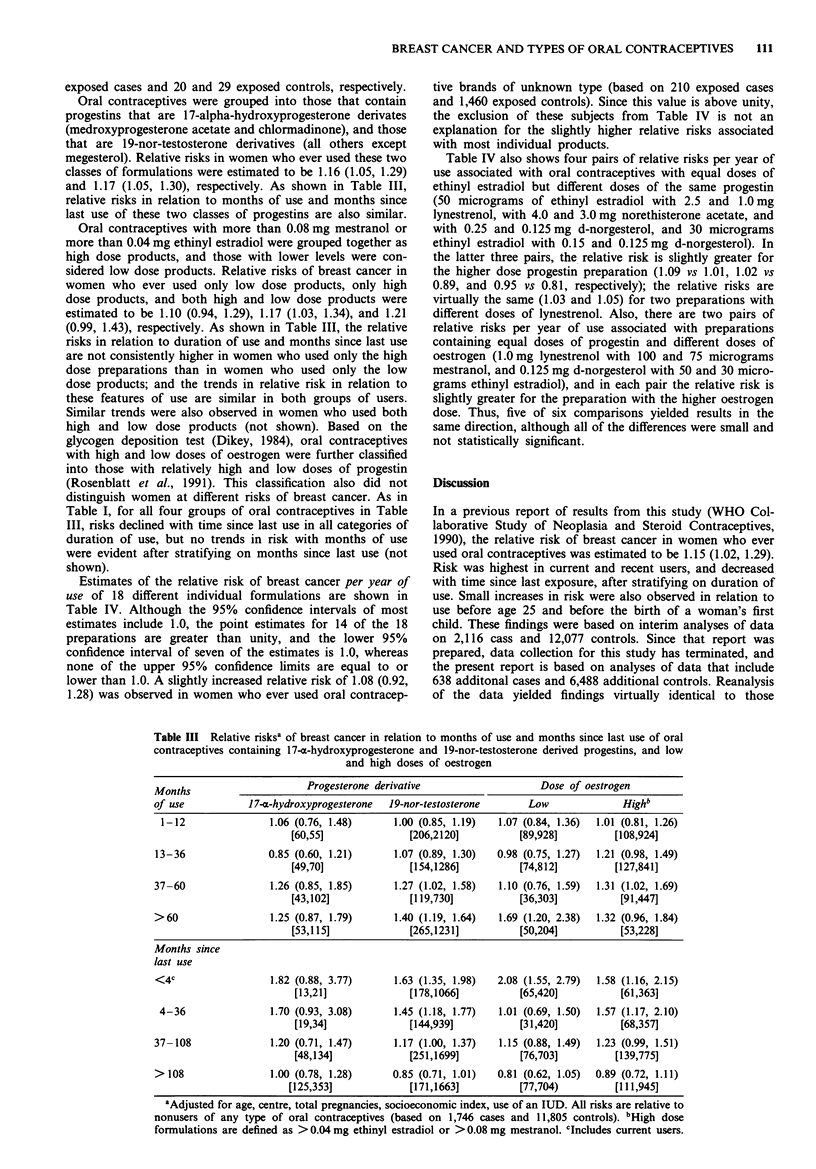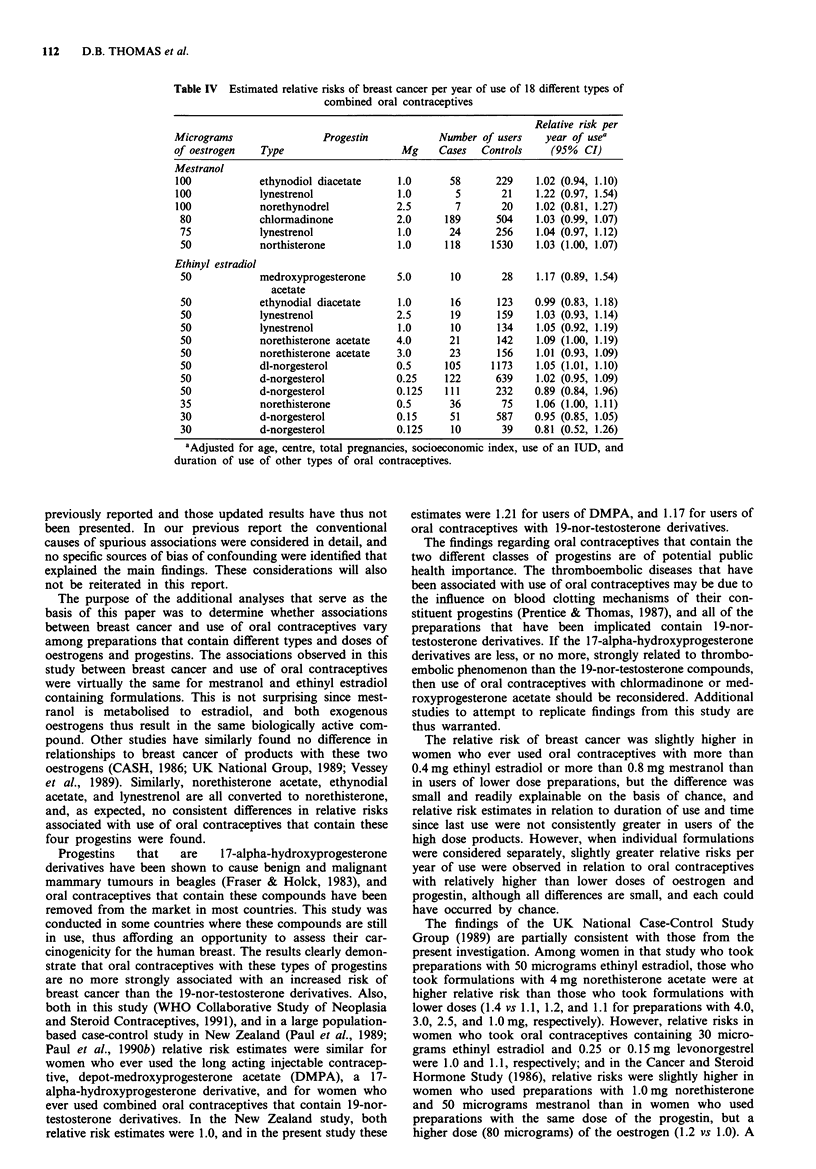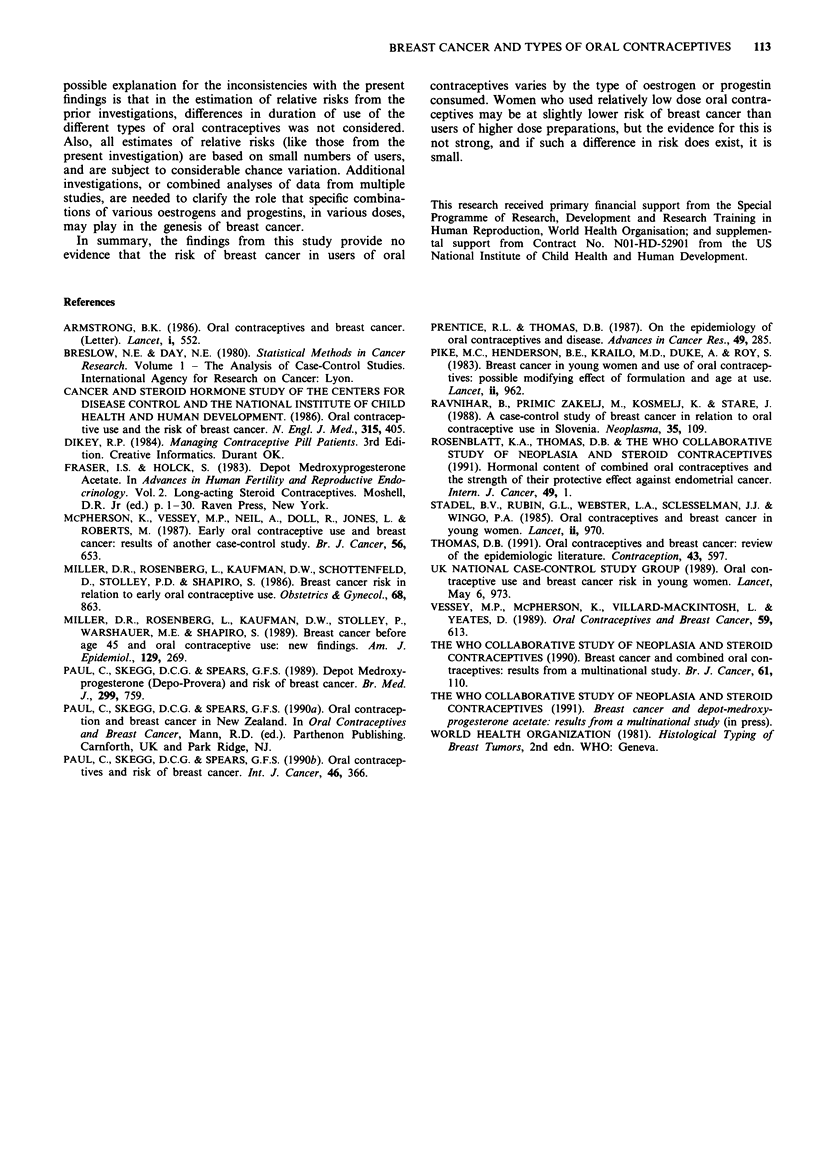# Breast cancer and specific types of combined oral contraceptives. The WHO Collaborative Study of Neoplasia and Steroid Contraceptives.

**DOI:** 10.1038/bjc.1992.20

**Published:** 1992-01

**Authors:** D. B. Thomas, E. A. Noonan

**Affiliations:** Fred Hutchinson Cancer Research Center, Seattle, WA 98104.

## Abstract

Data on 2,754 cases and 18,565 controls from a multinational hospital-based, case-control study were analysed to determine whether observed associations between combined oral contraceptives and breast cancer are similar for oral contraceptives with varying types and doses of oestrogens and progestins. After stratifying on duration of use, risk was found to be increased in current and recent users, and to decline with time since last use. These associations, of similar strength, were observed for users of products that contain mestranol and ethinyl estradiol, for women who used preparations with progestins derived from 19-nortestosterone and 17-alpha-hydroxyprogesterone, and for those who took preparations with relatively higher and lower doses of oestrogen. When products with equal doses of the same oestrogen or progestin and varying doses of the other hormonal constituent were considered, slightly higher relative risks per year of use were estimated for users of products with relatively higher than lower doses of either the constituent oestrogen or progestin, but the differences in relative risk could readily have occurred by chance. This study provides no evidence that risk of breast cancer in users of oral contraceptives varies by the type of oestrogen or progestin consumed.


Br. J. Cancer (1992), 65, 108-113 © Macmillan Press Ltd., 1992

### Breast cancer and specific types of combined oral contraceptives

D.B. Thomas, E.A. Noonan, and the WHO Collaborative Study of Neoplasia and Steroid Contraceptives*

**Summary** Data on 2,754 cases and 18,565 controls from a multinational hospital-based, case-control study were analysed to determine whether observed associations between combined oral contraceptives and breast cancer are similar for oral contraceptives with varying types and doses of oestrogens and progestins. After stratifying on duration of use, risk was found to be increased in current and recent users, and to decline with time since last use. These associations, of similar strength, were observed for users of products that contain mestranol and ethinyl estradiol, for women who used preparations with progestins derived from 19-nor-testosterone and 17-alpha-hydroxyprogesterone, and for those who took preparations with relatively higher and lower doses of oestrogen. When products with equal doses of the same oestrogen or progestin and varying doses of the other hormonal constituent were considered, slightly higher relative risks per year of use were estimated for users of products with relatively higher than lower doses of either the constituent oestrogen or progestin, but the differences in relative risk could readily have occurred by chance. This study provides no evidence that risk of breast cancer in users of oral contraceptives varies by the type of oestrogen or progestin consumed.

Correspondence: D.B. Thomas, Fred Hutchinson Cancer Research Center, 1124 Columbia Street, Seattle, WA 98104, USA.

*The data collection centers, and the principal investigator (PI), co-investigator (CI), and pathologist (P) at each participating center in alphabetical order by country, are as follows:
*University of Sydney*, Department of Public Health, Sydney, Australia: Geoffrey Berry (PI), Robert MacLennan (CI), Rodney Shearman (CI), Tatiana Jelihovsky (P), Joan Cooper Booth (P).
*University of Chile*, Faculty of Medicine, Hospital Jose Joaquin Aguirre, Department of Obstetrics and Gynecology, and the Ministry of Health, Hospital Salvador, Department Obstetrics and Gynecology, Santiago, Chile: Ramiro Molina (PI), Luis Martinez (CI), Oriana Salas (CI), Alfredo Dabancens (P).
*Shanghai Institute of Planned Parenthood Research*, Shanghai, China: Chen Zhiheng (PI), Tao Yun (CI), Hu Yong Wei (P).
*Hospital Universitario*, WHO Collaborative Center for Research in Human Reproduction, Cali, Colombia: Alvaro Cuadros (PI), Nubia Aristizabal (P).
*Central Institute of Cancer Research, Academy of Sciences of the German Democratic Republic*, Berlin, Germany: K. Ebeling (P), P. Nishan (CI), D. Kunde (P).
*Chiam Sheba Medical Center*, Department of Clinical Epidemiology, Tel Hashomer, Israel: Baruch Modan (PI), Elaine Ron (CI), Esther Alfandary (CI).
*University of Nairobi*, Nairobi Center for Research in Reproduction, Nairobi, Kenya: J.G. Mati (PI), Patrick Kenya (CI), Alfred Kungu (P), D. Gatei (P).
*Hospital General de Mexico*, Mexico City: Hector Rodriguez Cuevas (PI), Socorro Benavides Salazar (CI), Antonio Palet (P), Patricia Ontiveros (P).
*University of the Philippines*, College of Medicine, Manila, Philippines: Ruben A. Apelo (PI), Julietta R. de la Cruz (CI), Jose Baens (CI), Benita Javier (P).
*Chiang Mai University*, Faculty of Medicine, Chiang Mai, Thailand: Suporn Silpisornkosol (PI), Tieng Pardthaisong (CI), Nimit Martin (CI), Choti Theetranont (P).
*Chulalongkorn University*, Faculty of Medicine, Department of Obstetrics and Gynecology, WHO Collaborating Centre for Research in Human Reproduction, Bangkok, Thailand: Banpot Boosiri (PI), Supawat Chutivongse (PI), Pramuan Virutamasen (CI), Chansuda Wongsrichanalai (CI), Prasarn Jimakorn (P).
*Mahidol University*, Faculty of Medicine, Siriraj Hospital, Department of Obstetrics and Gynaecology, Family Planning Research Unit, Bangkok, Thailand: Suporn Koetsawang (PI), Daungdao Rachawat (CI), Nivat Chantarakul (P).
*University of Tromsø*, Institute of Medical Biology, Tromsø, Norway: Helge Stalsberg (Reference Pathologist).
*Fred Hutchinson Cancer Research Center*, Division of Public Health Sciences, Seattle, Washington, United States of America; Coordinating Center: David B. Thomas (Study Coordinator), Elizabeth A. Noonan. (Statician).
*World Health Organisation*, Olav Meirik and Timothy M.M. Farley, Special Programme of Research, Development and Research Training in Human Reproduction, Geneva, Switzerland.
Received 4 June 1991; and in revised form 3 September 1991.

The possible influence of combined oral contraceptive use on risk of breast cancer has been studied extensively during the past two decades, and the existing evidence has been reviewed on multiple occasions (e.g., Thomas, 1991). Results of epidemiologic studies have yielded inconsistent results, and it cannot be concluded with certainty that these hormonal preparations alter risk of breast cancer. If they do, the observed effect has been small.

Most studies have considered combined oral contraceptives to be a single entity, but they are actually a heterogeneous group of products that vary in both type and dose of oestrogen and progestin, and it is reasonable to expect that their influence on risk of breast cancer would not be uniform. Differences in the products under study could therefore be an explanation for the inconsistent results among studies; and the observation of only small increases in risk could be a result of those formulations that are carcinogenic for the human breast constituting a small proportion of all oral contraceptives.

The attempts that have been made to assess possible associations between breast cancer and specific types or groups of combined oral contraceptives have not yielded consistent results. In 1983, Pike *et al.* observed a particularly strong association between breast cancer and use before the age of 25 years of oral contraceptives with high progestin potency as determined by the delay of menses test; but this was not confirmed in two other studies (Stadel *et al.*, 1985; Miller *et al.*, 1986), and the validity of the classification that Pike used has been questioned (Armstrong, 1986). Two studies have failed to show associations between breast cancer and use at any time of oral contraceptives with specific types of progestins (CASH, 1986; Vessey *et al.*, 1989).

McPherson *et al.* (1987) reported an increase in risk of breast cancer with duration of use before the birth of a woman's first child of oral contraceptives that contain ethinyl estradiol, but not that contain mestranol; but this was not confirmed in two subsequent studies (Vessey *et al.*, 1989; Paul *et al.*, 1990*a*). Also, no differences in relative risks in relation to overall duration of use of preparations with ethinyl estradiol and mestranol were observed in three separate studies (CASH, 1986; UK National, 1989; Vessey *et al.*, 1989). Similarly, no consistent increases in risk in relation to specific types of oral contraceptives were observed among five different investigations (CASH, 1986; Miller *et al.*, 1989; UK National Group, 1989; Vessey, 1989; Ravnihar *et al.*, 1988). In addition, relative risks were not consistently greater in relation to individual formulations with high doses of oestrogen than in relation to those with lower doses, although in the UK National Case-Control Study (1989), a higher relative risk was observed in women who had used oral contraceptives with more than 50 micrograms of oestrogen (either ethinyl estradiol or mestranol) for over 8 years than in women who had used lower dose preparations for the same length of time.

The studies to date have reported results largely in relation to use of oral contraceptives that contain progestins that are derivatives of 19-nor-testosterone. None have studied oral contraceptives that contain derivatives of 17-alpha hydroxy-progesterone (i.e., medroxyprogesterone acetate and chloramadinone); and it is these progestins that have been shown to cause breast cancer in beagles (Fraser & Holck, 1983). Also, estimates of relative risks of breast cancer in relation to the newer lower dose oral contraceptives have not been compared with estimates for the older, higher dose preparations. Data from this multinational study include information on use of a wide range of formulations, and provide an opportunity to address these two issues. In addition, we have attempted to confirm the results of previous studies, and present results of analyses to assess associations between breast cancer and specific individual oral contraceptives, and oral contraceptives with specific types of oestrogens and progestins.

### Methods

The methods used in this study have been previously described (WHO Collaborative Study of Neoplasia and Steroid Contraceptives, 1990). Data were collected in 12 participating centres in Australia, Chile, the Peoples Republic of China, Colombia, The German Democratic Republic (GDR), Israel, Kenya, Mexico, the Philippines, and Thailand. Data were collected from thee separate centres in Thailand (Siriraj and Chulalongkorn in Bangkok and Chiang Mai). Data collection began in the various centres between October 1979 and November 1982 and ceased between September 1984 and September 1988.

In each hospital, cases were detected by monitoring all new admissions to wards where women with breast cancer were treated, and by checking outpatient gynecological and tumour clinics, and records of hospital pathology laboratories. Cases included all women diagnosed locally as having a malignant breast tumour, born either after 1924 or after 1929 (depending on when oral contraceptives were first locally available), and who resided during the preceding year in a defined geographic area served by the hospital.

Controls were selected from among women admitted to other than obstetric and gynecologic wards, who met the same age and residential criteria for eligibility as the cases, and who were not admitted for treatment of conditions considered *a priori* to possibly alter contraceptive practices (i.e., circulatory and cardiovascular diseases, diabetes, chronic renal disease, benign breast disease, a previously diagnosed malignancy, chronic liver disease, and any obstetric or gynecologic condition).

Approximately two controls were selected for each case, but controls were not matched to individual cases. As this was a study of cancers in addition to those of the breast (i.e., cervix and corpus uteri, ovary, and liver), more than two controls per breast cancer case were available for analysis.

A standardised questionnaire was administered in person to all study subjects to obtain information on the known and suspected risk factors for the neoplasms under study, and a complete obstetric and contraceptive history. A calendar and samples of locally available oral contraceptives were used to facilitate recall of times of use and products taken. In addition, the medical records of women who gave a history of oral contraceptive use were reviewed when available, and in such instances information from both interviews and these records were utilised by the interviewers to record details of the woman's use. The questionnaire was printed in the local language in all countries except Kenya where multiple languages are spoken, and the Philippines and Australia, where English is widely used. Where the information was not recorded directly on the English version questionnaire, it was transcribed onto an English version for mailing to the coordinating centre in Seattle.

Pathologists at each centre were responsible for provisional histological diagnosis of the cases. Slides from all cases were sent to a single reference pathologist for confirmation of diagnosis and uniform histologic classification according to the WHO histologic classification of breast tumours (World Health Organisation, 1981). Only data from cases considered by the reference pathologist to have invasive carcinoma of the breast were utilised for this report.

Of the 2,996 eligible cases and 20,216 controls selected for this study, 2,835 (94.6%) and 19,221 (95.1%) were interviewed, respectively. As described subsequently, estimates of relative risks in users of oral contraceptives were controlled for variables that appeared to confound the relationship between breast cancer and these products. Eighty-one cases and 656 controls were excluded from the analyses because values for one or more of the identified confounders were missing or the duration of al episodes of oral contraceptive use was unknown. Of the remaining 2,754 cases, 70.1%, 10.8% and 4.9% had carcinomas classified as ductal, lobular, and apocrine, respectively; the remaining 14.2% had one of 15 other histologic types.

Since cases tended to be older than controls, and since both the ratio of controls to cases and the prevalence of use of oral contraceptives varied among the centres, all relative risk estimates were controlled for age and centre. Unconditional logistic regression analyses (Breslow & Day, 1980, pages 192–246) were utilised to estimate relative risks, adjusted for these and other potentially confounding variables. These variables were entered into the regression models as categorical variables. To control for multiple factors simultaneously, a final model containing confounding variables was constructed. Variables were entered into models sequentially, one at a time, and retained if the associated chi-square test for goodness of fit was significant ($P < 0.05$) and if the resultant relative risk in relation to ever use oral contraceptives was altered by more than 5%. This model was then used to estimate relative risks of breast cancer and their 95% confidence intervals in users of various types of oral contraceptives.

To assess possible relationships of individual formulations to risk of breast cancer, months of use of the specific oral contraceptive under consideration, and total months of use of all other types combined, were included in logistic models as single continuous variables, and relative risks per year of use were estimated. Although time since first use was more strongly related to risk of breast cancer than duration of use, these two features of exposure were strongly correlated in our data, and relative risk for year of use thus provides a valid index by which individual types of oral contraceptives can be compared, that makes maximum use of the information on each episode of use.

### Results

As reported in a previous publication (WHO Collaborative Study of Neoplasia and Steroid Contraceptives, 1990), risk was observed to be increased in current and recent users of oral contraceptives, and to decline with time since last use in all categories of duration, but no trend with months of use was observed after stratifying on months since last use. As shown in Table I, these findings were also equally evident in users of mestranol and ethinyl oestradiol containing oral contraceptives and the relative risk estimates are not consistently higher for one class of preparations than for the other.

Relative risks in women who used oral contraceptives before the birth of their first child for more than 1 year were estimated to be 2.06 (1.01, 4.17) and 1.19 (0.62, 2.31) in users of mestranol and ethinyl oestradiol containing products, respectively. These values are based on small numbers of users, and their difference could have occurred by chance.

Relative risks in women who used mestranol and ethinyl oestradiol containing formulations before age 25 were estimated respectively, to be 1.05 (0.82, 1.34) and 1.10 (0.88, 1.36) for ever-users, and 1.78 (1.00, 3.19) and 1.81 (1.15, 2.84) for women who used these products for over 3 years before age 25. Relative risk estimates did not vary significantly among women who had used individual formulations before age 25, but all such estimates were based in small numbers of users and had wide confidence limits.

Table II shows relative risks in relation to duration of use and months since last use of oral contraceptives that contain various types of progestins. Relative risks tend to increase with duration of use of all progestins shown except ethynodial diacetate, and to decrease with time since last exposure to all progestin types. Small numbers of users precluded simultaneous consideration of these two features of use. There were too few users of oral contraceptives that contain norethynodrel and medroxyprogesterone acetate to allow estimates of relative risks in relation to duration or recency of their use; relative risks in women who ever used oral contraceptives with these progestins were estimated to be 0.94 (0.38, 2.32) and 1.30 (0.66, 2.57), based on seven and 13

**Table I** Relative risks[a] of breast cancer in relation to months of use and months since last use of oral contraceptives containing mestranol and ethinyl estradiol

| *Months since last use* | *Months of use* 1–12 | 13–36 | 37–84 | >84 | *All users* |
|---|---|---|---|---|---|
| | | | *ethinyl estradiol* | | |
| ≤3[b] | 1.06 (0.55, 2.07) [10,163] | 1.12 (0.66, 1.90) [17,194] | 1.78 (1.24, 2.55) [45,200] | 1.52 (1.12, 2.07) [67,203] | 1.46 (1.18, 1.82) [139,760] |
| 4–36 | 1.83 (1.19, 2.81) [29,224] | 1.37 (0.85, 2.21) [23,160] | 1.48 (0.98, 2.22) [33,151] | 1.34 (0.89, 2.03) [34,118] | 1.48 (1.18, 1.86) [119,653] |
| 37–108 | 0.81 (0.58, 1.14) [42,468] | 1.29 (0.92, 1.80) [48,269] | 1.36 (1.03, 1.80) [75,303] | 1.28 (0.82, 1.99) [28,114] | 1.15 (0.96, 1.38) [193,1154] |
| >108 | 0.92 (0.71, 1.20) [70,618] | 0.87 (0.61, 1.25) [37,319] | 1.00 (0.63, 1.58) [23,161] | 1.00 (0.23, 4.34) [2,20] | 0.92 (0.75, 1.12) [132,1118] |
| All times | 0.98 (0.81, 1.19) [151,1473] | 1.10 (0.90, 1.36) [925,942] | 1.38 (1.14, 1.68) [176,815] | 1.39 (1.11, 1.74) [131,455] | 1.18 (1.04, 1.33) [583,3685] |
| | | | *mestranol* | | |
| <4[b] | 3.31 (1.32, 8.33) [6,48] | 1.41 (0.48, 4.12) [4,48] | 1.34 (0.46, 3.87) [4,44] | 1.75 (0.92, 3.34) [13,62] | 1.70 (1.23, 2.35) [27,202] |
| 4–36 | 1.29 (0.70, 2.39) [13,147] | 1.62 (0.80, 3.29) [10,80] | 0.72 (0.28, 1.85) [5,73] | 2.72 (1.42, 5.24) [14,42] | 1.45 (1.02, 2.06) [42,342] |
| 37–108 | 1.36 (0.97, 1.89) [47,378] | 0.89 (0.54, 1.46) [19,203] | 1.66 (1.13, 2.44) [38,187] | 1.76 (1.03, 2.99) [20,82] | 1.34 (1.07, 1.66) [124,850] |
| >108 | 0.95 (0.73, 1.23) [81,586] | 0.90 (0.67, 1.21) [63,343] | 1.01 (0.74, 1.36) [63,289] | 1.05 (0.64, 1.73) [21,284] | 0.90 (0.75, 1.08) [228,1342] |
| All times | 1.13 (0.93, 1.37) [147,1159] | 0.97 (0.76, 1.24) [96,674] | 1.17 (0.93, 1.48) [110,593] | 1.58 (1.18, 2.11) [68,310] | 1.15 (1.00, 1.32) [421,2736] |

[a]Adjusted for age, centre, total pregnancies, socioeconomic index, use of an IUD. All risks are relative to nonusers of any type of oral contraceptives (based on 1,746 cases and 11,805 controls). 95% confidence intervals are in ( ), and number of cases and controls are in [ ]. [b]Includes current users.

**Table II** Relative risks[a] of breast cancer in relation to months of use and months since last use of oral contraceptives containing various progestins

| *Months of use* | *Chlormadinone acetate* | *Norethisterone acetate* | *Norethisterone* | *Ethynodial diacetate* | *Lynestrenol* | *DL-norgestrol* |
|---|---|---|---|---|---|---|
| 1–12 | 1.01 (0.71, 1.44) [54,150] | 1.03 (0.75, 1.42) [58,275] | 1.04 (0.79, 1.37) [63,851] | 1.50 (1.04, 2.17) [42,184] | 0.79 (0.48, 1.28) [19,280] | 1.06 (0.86, 1.32) [117,1170] |
| 13–36 | 0.72 (0.49, 1.05) [45,161] | 1.02 (0.74, 1.42) [57,229] | 1.18 (0.82, 1.69) [37,405] | 0.74 (0.43, 1.29) [16,114] | 0.73 (0.38, 1.39) [11,149] | 1.29 (1.01, 1.64) [98,703] |
| 37–60/>37 | 1.10 (0.73, 1.66) [41,97] | 1.19 (0.83, 1.70) [50,152] | 1.50 (1.15, 1.96) [76,544] | 1.10 (0.66, 1.83) [21,92] | 1.45 (0.95, 2.22) [28,180] | 1.30 (1.04, 1.61) [127,738] |
| >60 | 1.12 (0.78, 1.62) [42,115] | 1.17 (0.88, 1.55) [85,246] | | | | |
| *Months since last use* | | | | | | |
| <4 | 2.14 (0.77, 5.93) [7,9] | 1.63 (1.09, 2.44) [40,96] | 2.06 (1.45, 2.93) [43,300] | 2.04 (0.75, 5.51) [6,23] | 1.93 (1.00, 3.71) [11,102] | 1.30 (0.99, 1.70) [77,552] |
| 4–36 | 1.95 (0.95, 4.02) [14,20] | 1.27 (1.09, 2.44) [36,117] | 1.14 (0.74, 1.76) [24,339] | 1.85 (0.93, 3.68) [12,47] | 1.69 (0.90, 3.17) [13,90] | 1.57 (1.20, 2.05) [79,500] |
| 37–108 | 0.95 (0.62, 1.47) [32,95] | 1.10 (0.85, 1.42) [107,350] | 1.27 (0.95, 1.70) [59,607] | 1.10 (0.71, 1.70) [28,135] | 1.22 (0.75, 1.98) [21,166] | 1.28 (1.02, 1.59) [199,855] |
| >108 | 1.01 (0.79, 1.29) [139,389] | 1.08 (0.81, 1.44) [67,339] | 0.94 (0.69, 1.28) [48,554] | 1.07 (0.72, 1.59) [33,185] | 0.50 (0.28, 0.89) [13,251] | 0.98 (0.75, 1.28) [67,704] |

[a]Adjusted for age, centre, total pregnancies, socioeconomic index, use of an IUD. All risks are relative to nonusers of any type of oral contraceptives (based on 1,746 cases and 11,805 controls). 95% confidence intervals are in ( ), and number of cases and controls are in [ ].

exposed cases and 20 and 29 exposed controls, respectively.

Oral contraceptives were grouped into those that contain progestins that are 17-alpha-hydroxyprogesterone derivates (medroxyprogesterone acetate and chlormadinone), and those that are 19-nor-testosterone derivatives (all others except megesterol). Relative risks in women who ever used these two classes of formulations were estimated to be 1.16 (1.05, 1.29) and 1.17 (1.05, 1.30), respectively. As shown in Table III, relative risks in relation to months of use and months since last use of these two classes of progestins are also similar.

Oral contraceptives with more than 0.08 mg mestranol or more than 0.04 mg ethinyl estradiol were grouped together as high dose products, and those with lower levels were considered low dose products. Relative risks of breast cancer in women who ever used only low dose products, only high dose products, and both high and low dose products were estimated to be 1.10 (0.94, 1.29), 1.17 (1.03, 1.34), and 1.21 (0.99, 1.43), respectively. As shown in Table III, the relative risks in relation to duration of use and months since last use are not consistently higher in women who used only the high dose preparations than in women who used only the low dose products; and the trends in relative risk in relation to these features of use are similar in both groups of users. Similar trends were also observed in women who used both high and low dose products (not shown). Based on the glycogen deposition test (Dikey, 1984), oral contraceptives with high and low doses of oestrogen were further classified into those with relatively high and low doses of progestin (Rosenblatt *et al.*, 1991). This classification also did not distinguish women at different risks of breast cancer. As in Table I, for all four groups of oral contraceptives in Table III, risks declined with time since last use in all categories of duration of use, but no trends in risk with months of use were evident after stratifying on months since last use (not shown).

Estimates of the relative risk of breast cancer *per year of use* of 18 different individual formulations are shown in Table IV. Although the 95% confidence intervals of most estimates include 1.0, the point estimates for 14 of the 18 preparations are greater than unity, and the lower 95% confidence interval of seven of the estimates is 1.0, whereas none of the upper 95% confidence limits are equal to or lower than 1.0. A slightly increased relative risk of 1.08 (0.92, 1.28) was observed in women who ever used oral contraceptive brands of unknown type (based on 210 exposed cases and 1,460 exposed controls). Since this value is above unity, the exclusion of these subjects from Table IV is not an explanation for the slightly higher relative risks associated with most individual products.

Table IV also shows four pairs of relative risks per year of use associated with oral contraceptives with equal doses of ethinyl estradiol but different doses of the same progestin (50 micrograms of ethinyl estradiol with 2.5 and 1.0 mg lynestrenol, with 4.0 and 3.0 mg norethisterone acetate, and with 0.25 and 0.125 mg d-norgesterol, and 30 micrograms ethinyl estradiol with 0.15 and 0.125 mg d-norgesterol). In the latter three pairs, the relative risk is slightly greater for the higher dose progestin preparation (1.09 *vs* 1.01, 1.02 *vs* 0.89, and 0.95 *vs* 0.81, respectively); the relative risks are virtually the same (1.03 and 1.05) for two preparations with different doses of lynestrenol. Also, there are two pairs of relative risks per year of use associated with preparations containing equal doses of progestin and different doses of oestrogen (1.0 mg lynestrenol with 100 and 75 micrograms mestranol, and 0.125 mg d-norgesterol with 50 and 30 micrograms ethinyl estradiol), and in each pair the relative risk is slightly greater for the preparation with the higher oestrogen dose. Thus, five of six comparisons yielded results in the same direction, although all of the differences were small and not statistically significant.

### Discussion

In a previous report of results from this study (WHO Collaborative Study of Neoplasia and Steroid Contraceptives, 1990), the relative risk of breast cancer in women who ever used oral contraceptives was estimated to be 1.15 (1.02, 1.29). Risk was highest in current and recent users, and decreased with time since last exposure, after stratifying on duration of use. Small increases in risk were also observed in relation to use before age 25 and before the birth of a woman's first child. These findings were based on interim analyses of data on 2,116 cass and 12,077 controls. Since that report was prepared, data collection for this study has terminated, and the present report is based on analyses of data that include 638 additonal cases and 6,488 additional controls. Reanalysis of the data yielded findings virtually identical to those

**Table III** Relative risks[a] of breast cancer in relation to months of use and months since last use of oral contraceptives containing 17-α-hydroxyprogesterone and 19-nor-testosterone derived progestins, and low and high doses of oestrogen

| *Months of use* | *Progesterone derivative* | | *Dose of oestrogen* | |
|---|---|---|---|---|
| | *17-α-hydroxyprogesterone* | *19-nor-testosterone* | *Low* | *High*[b] |
| 1–12 | 1.06 (0.76, 1.48) [60,55] | 1.00 (0.85, 1.19) [206,2120] | 1.07 (0.84, 1.36) [89,928] | 1.01 (0.81, 1.26) [108,924] |
| 13–36 | 0.85 (0.60, 1.21) [49,70] | 1.07 (0.89, 1.30) [154,1286] | 0.98 (0.75, 1.27) [74,812] | 1.21 (0.98, 1.49) [127,841] |
| 37–60 | 1.26 (0.85, 1.85) [43,102] | 1.27 (1.02, 1.58) [119,730] | 1.10 (0.76, 1.59) [36,303] | 1.31 (1.02, 1.69) [91,447] |
| >60 | 1.25 (0.87, 1.79) [53,115] | 1.40 (1.19, 1.64) [265,1231] | 1.69 (1.20, 2.38) [50,204] | 1.32 (0.96, 1.84) [53,228] |
| *Months since last use* | | | | |
| <4[c] | 1.82 (0.88, 3.77) [13,21] | 1.63 (1.35, 1.98) [178,1066] | 2.08 (1.55, 2.79) [65,420] | 1.58 (1.16, 2.15) [61,363] |
| 4–36 | 1.70 (0.93, 3.08) [19,34] | 1.45 (1.18, 1.77) [144,939] | 1.01 (0.69, 1.50) [31,420] | 1.57 (1.17, 2.10) [68,357] |
| 37–108 | 1.20 (0.71, 1.47) [48,134] | 1.17 (1.00, 1.37) [251,1699] | 1.15 (0.88, 1.49) [76,703] | 1.23 (0.99, 1.51) [139,775] |
| >108 | 1.00 (0.78, 1.28) [125,353] | 0.85 (0.71, 1.01) [171,1663] | 0.81 (0.62, 1.05) [77,704] | 0.89 (0.72, 1.11) [111,945] |

[a]Adjusted for age, centre, total pregnancies, socioeconomic index, use of an IUD. All risks are relative to nonusers of any type of oral contraceptives (based on 1,746 cases and 11,805 controls). [b]High dose formulations are defined as >0.04 mg ethinyl estradiol or >0.08 mg mestranol. [c]Includes current users.

**Table IV** Estimated relative risks of breast cancer per year of use of 18 different types of combined oral contraceptives

| *Micrograms of oestrogen* | *Progestin Type* | *Mg* | *Number of users Cases* | *Controls* | *Relative risk per year of use*[a] *(95% CI)* |
|---|---|---|---|---|---|
| *Mestranol* | | | | | |
| 100 | ethynodiol diacetate | 1.0 | 58 | 229 | 1.02 (0.94, 1.10) |
| 100 | lynestrenol | 1.0 | 5 | 21 | 1.22 (0.97, 1.54) |
| 100 | norethynodrel | 2.5 | 7 | 20 | 1.02 (0.81, 1.27) |
| 80 | chlormadinone | 2.0 | 189 | 504 | 1.03 (0.99, 1.07) |
| 75 | lynestrenol | 1.0 | 24 | 256 | 1.04 (0.97, 1.12) |
| 50 | northisterone | 1.0 | 118 | 1530 | 1.03 (1.00, 1.07) |
| *Ethinyl estradiol* | | | | | |
| 50 | medroxyprogesterone acetate | 5.0 | 10 | 28 | 1.17 (0.89, 1.54) |
| 50 | ethynodial diacetate | 1.0 | 16 | 123 | 0.99 (0.83, 1.18) |
| 50 | lynestrenol | 2.5 | 19 | 159 | 1.03 (0.93, 1.14) |
| 50 | lynestrenol | 1.0 | 10 | 134 | 1.05 (0.92, 1.19) |
| 50 | norethisterone acetate | 4.0 | 21 | 142 | 1.09 (1.00, 1.19) |
| 50 | norethisterone acetate | 3.0 | 23 | 156 | 1.01 (0.93, 1.09) |
| 50 | dl-norgesterol | 0.5 | 105 | 1173 | 1.05 (1.01, 1.10) |
| 50 | d-norgesterol | 0.25 | 122 | 639 | 1.02 (0.95, 1.09) |
| 50 | d-norgesterol | 0.125 | 111 | 232 | 0.89 (0.84, 1.96) |
| 35 | norethisterone | 0.5 | 36 | 75 | 1.06 (1.00, 1.11) |
| 30 | d-norgesterol | 0.15 | 51 | 587 | 0.95 (0.85, 1.05) |
| 30 | d-norgesterol | 0.125 | 10 | 39 | 0.81 (0.52, 1.26) |

[a]Adjusted for age, centre, total pregnancies, socioeconomic index, use of an IUD, and duration of use of other types of oral contraceptives.

previously reported and those updated results have thus not been presented. In our previous report the conventional causes of spurious associations were considered in detail, and no specific sources of bias of confounding were identified that explained the main findings. These considerations will also not be reiterated in this report.

The purpose of the additional analyses that serve as the basis of this paper was to determine whether associations between breast cancer and use of oral contraceptives vary among preparations that contain different types and doses of oestrogens and progestins. The associations observed in this study between breast cancer and use of oral contraceptives were virtually the same for mestranol and ethinyl estradiol containing formulations. This is not surprising since mestranol is metabolised to estradiol, and both exogenous oestrogens thus result in the same biologically active compound. Other studies have similarly found no difference in relationships to breast cancer of products with these two oestrogens (CASH, 1986; UK National Group, 1989; Vessey *et al.*, 1989). Similarly, norethisterone acetate, ethynodial acetate, and lynestrenol are all converted to norethisterone, and, as expected, no consistent differences in relative risks associated with use of oral contraceptives that contain these four progestins were found.

Progestins that are 17-alpha-hydroxyprogesterone derivatives have been shown to cause benign and malignant mammary tumours in beagles (Fraser & Holck, 1983), and oral contraceptives that contain these compounds have been removed from the market in most countries. This study was conducted in some countries where these compounds are still in use, thus affording an opportunity to assess their carcinogenicity for the human breast. The results clearly demonstrate that oral contraceptives with these types of progestins are no more strongly associated with an increased risk of breast cancer than the 19-nor-testosterone derivatives. Also, both in this study (WHO Collaborative Study of Neoplasia and Steroid Contraceptives, 1991), and in a large population-based case-control study in New Zealand (Paul *et al.*, 1989; Paul *et al.*, 1990*b*) relative risk estimates were similar for women who ever used the long acting injectable contraceptive, depot-medroxyprogesterone acetate (DMPA), a 17-alpha-hydroxyprogesterone derivative, and for women who ever used combined oral contraceptives that contain 19-nortestosterone derivatives. In the New Zealand study, both relative risk estimates were 1.0, and in the present study these estimates were 1.21 for users of DMPA, and 1.17 for users of oral contraceptives with 19-nor-testosterone derivatives.

The findings regarding oral contraceptives that contain the two different classes of progestins are of potential public health importance. The thromboembolic diseases that have been associated with use of oral contraceptives may be due to the influence on blood clotting mechanisms of their constituent progestins (Prentice & Thomas, 1987), and all of the preparations that have been implicated contain 19-nortestosterone derivatives. If the 17-alpha-hydroxyprogesterone derivatives are less, or no more, strongly related to thromboembolic phenomenon than the 19-nor-testosterone compounds, then use of oral contraceptives with chlormadinone or medroxyprogesterone acetate should be reconsidered. Additional studies to attempt to replicate findings from this study are thus warranted.

The relative risk of breast cancer was slightly higher in women who ever used oral contraceptives with more than 0.4 mg ethinyl estradiol or more than 0.8 mg mestranol than in users of lower dose preparations, but the difference was small and readily explainable on the basis of chance, and relative risk estimates in relation to duration of use and time since last use were not consistently greater in users of the high dose products. However, when individual formulations were considered separately, slightly greater relative risks per year of use were observed in relation to oral contraceptives with relatively higher than lower doses of oestrogen and progestin, although all differences are small, and each could have occurred by chance.

The findings of the UK National Case-Control Study Group (1989) are partially consistent with those from the present investigation. Among women in that study who took preparations with 50 micrograms ethinyl estradiol, those who took formulations with 4 mg norethisterone acetate were at higher relative risk than those who took formulations with lower doses (1.4 *vs* 1.1, 1.2, and 1.1 for preparations with 4.0, 3.0, 2.5, and 1.0 mg, respectively). However, relative risks in women who took oral contraceptives containing 30 micrograms ethinyl estradiol and 0.25 or 0.15 mg levonorgestrel were 1.0 and 1.1, respectively; and in the Cancer and Steroid Hormone Study (1986), relative risks were slightly higher in women who used preparations with 1.0 mg norethisterone and 50 micrograms mestranol than in women who used preparations with the same dose of the progestin, but a higher dose (80 micrograms) of the oestrogen (1.2 *vs* 1.0). A

possible explanation for the inconsistencies with the present findings is that in the estimation of relative risks from the prior investigations, differences in duration of use of the different types of oral contraceptives was not considered. Also, all estimates of relative risks (like those from the present investigation) are based on small numbers of users, and are subject to considerable chance variation. Additional investigations, or combined analyses of data from multiple studies, are needed to clarify the role that specific combinations of various oestrogens and progestins, in various doses, may play in the genesis of breast cancer.

In summary, the findings from this study provide no evidence that the risk of breast cancer in users of oral contraceptives varies by the type of oestrogen or progestin consumed. Women who used relatively low dose oral contraceptives may be at slightly lower risk of breast cancer than users of higher dose preparations, but the evidence for this is not strong, and if such a difference in risk does exist, it is small.

This research received primary financial support from the Special Programme of Research, Development and Research Training in Human Reproduction, World Health Organisation; and supplemental support from Contract No. N01-HD-52901 from the US National Institute of Child Health and Human Development.

### References

ARMSTRONG, B.K. (1986). Oral contraceptives and breast cancer. (Letter). *Lancet*, **i**, 552.

BRESLOW, N.E. & DAY, N.E. (1980). *Statistical Methods in Cancer Research*. Volume 1 – The Analysis of Case-Control Studies. International Agency for Research on Cancer: Lyon.

CANCER AND STEROID HORMONE STUDY OF THE CENTERS FOR DISEASE CONTROL AND THE NATIONAL INSTITUTE OF CHILD HEALTH AND HUMAN DEVELOPMENT. (1986). Oral contraceptive use and the risk of breast cancer. *N. Engl. J. Med.*, **315**, 405.

DIKEY, R.P. (1984). *Managing Contraceptive Pill Patients*. 3rd Edition. Creative Informatics. Durant OK.

FRASER, I.S. & HOLCK, S. (1983). Depot Medroxyprogesterone Acetate. In *Advances in Human Fertility and Reproductive Endocrinology*. Vol. 2. Long-acting Steroid Contraceptives. Moshell, D.R. Jr (ed.) p. 1–30. Raven Press, New York.

MCPHERSON, K., VESSEY, M.P., NEIL, A., DOLL, R., JONES, L. & ROBERTS, M. (1987). Early oral contraceptive use and breast cancer: results of another case-control study. *Br. J. Cancer*, **56**, 653.

MILLER, D.R., ROSENBERG, L., KAUFMAN, D.W., SCHOTTENFELD, D., STOLLEY, P.D. & SHAPIRO, S. (1986). Breast cancer risk in relation to early oral contraceptive use. *Obstetrics & Gynecol.*, **68**, 863.

MILLER, D.R., ROSENBERG, L., KAUFMAN, D.W., STOLLEY, P., WARSHAUER, M.E. & SHAPIRO, S. (1989). Breast cancer before age 45 and oral contraceptive use: new findings. *Am. J. Epidemiol.*, **129**, 269.

PAUL, C., SKEGG, D.C.G. & SPEARS, G.F.S. (1989). Depot Medroxyprogesterone (Depo-Provera) and risk of breast cancer. *Br. Med. J.*, **299**, 759.

PAUL, C., SKEGG, D.C.G. & SPEARS, G.F.S. (1990*a*). Oral contraception and breast cancer in New Zealand. In *Oral Contraceptives and Breast Cancer*, Mann, R.D. (ed.). Parthenon Publishing. Carnforth, UK and Park Ridge, NJ.

PAUL, C., SKEGG, D.C.G. & SPEARS, G.F.S. (1990*b*). Oral contraceptives and risk of breast cancer. *Int. J. Cancer*, **46**, 366.

PRENTICE, R.L. & THOMAS, D.B. (1987). On the epidemiology of oral contraceptives and disease. *Advances in Cancer Res.*, **49**, 285.

PIKE, M.C., HENDERSON, B.E., KRAILO, M.D., DUKE, A. & ROY, S. (1983). Breast cancer in young women and use of oral contraceptives: possible modifying effect of formulation and age at use. *Lancet*, **ii**, 962.

RAVNIHAR, B., PRIMIC ZAKELJ, M., KOSMELJ, K. & STARE, J. (1988). A case-control study of breast cancer in relation to oral contraceptive use in Slovenia. *Neoplasma*, **35**, 109.

ROSENBLATT, K.A., THOMAS, D.B. & THE WHO COLLABORATIVE STUDY OF NEOPLASIA AND STEROID CONTRACEPTIVES (1991). Hormonal content of combined oral contraceptives and the strength of their protective effect against endometrial cancer. *Intern. J. Cancer*, **49**, 1.

STADEL, B.V., RUBIN, G.L., WEBSTER, L.A., SCLESSELMAN, J.J. & WINGO, P.A. (1985). Oral contraceptives and breast cancer in young women. *Lancet*, **ii**, 970.

THOMAS, D.B. (1991). Oral contraceptives and breast cancer: review of the epidemiologic literature. *Contraception*, **43**, 597.

UK NATIONAL CASE-CONTROL STUDY GROUP (1989). Oral contraceptive use and breast cancer risk in young women. *Lancet*, May 6, 973.

VESSEY, M.P., MCPHERSON, K., VILLARD-MACKINTOSH, L. & YEATES, D. (1989). *Oral Contraceptives and Breast Cancer*, **59**, 613.

THE WHO COLLABORATIVE STUDY OF NEOPLASIA AND STEROID CONTRACEPTIVES (1990). Breast cancer and combined oral contraceptives: results from a multinational study. *Br. J. Cancer*, **61**, 110.

THE WHO COLLABORATIVE STUDY OF NEOPLASIA AND STEROID CONTRACEPTIVES (1991). *Breast cancer and depot-medroxyprogesterone acetate: results from a multinational study* (in press).

WORLD HEALTH ORGANIZATION (1981). *Histological Typing of Breast Tumors*, 2nd edn. WHO: Geneva.